# Epigenetic regulation of *EFEMP1* in prostate cancer: biological relevance and clinical potential

**DOI:** 10.1111/jcmm.12394

**Published:** 2014-09-11

**Authors:** Mafalda Almeida, Vera L Costa, Natália R Costa, João Ramalho-Carvalho, Tiago Baptista, Franclim R Ribeiro, Paula Paulo, Manuel R Teixeira, Jorge Oliveira, Ragnhild A Lothe, Guro E Lind, Rui Henrique, Carmen Jerónimo

**Affiliations:** a Cancer Biology and Epigenetics Group, Research Center of the Portuguese Oncology Institute - PortoPorto, Portugal; b Department of Cancer Prevention, Institute for Cancer Research, The Norwegian Radium Hospital, Oslo University HospitalOslo, Norway; c Centre for Cancer Biomedicine, University of OsloOslo, Norway; d Cancer Genetics Group, Research Center of the Portuguese Oncology Institute - PortoPorto, Portugal; e Department of Genetics, Portuguese Oncology Institute - PortoPorto, Portugal; f Department of Pathology and Molecular Immunology, Institute of Biomedical Sciences Abel Salazar, University of PortoPorto, Portugal; g Department of Urology, Portuguese Oncology Institute - PortoPorto, Portugal; h Faculty of Medicine, University of OsloOslo, Norway; i Department of Pathology, Portuguese Oncology Institute - PortoPorto, Portugal

**Keywords:** DNA methylation, prostate cancer, *EFEMP1*, diagnosis, biomarker, histone post-translational modifications

## Abstract

Epigenetic alterations are common in prostate cancer (PCa) and seem to contribute decisively to its initiation and progression. Moreover, aberrant promoter methylation is a promising biomarker for non-invasive screening. Herein, we sought to characterize EFEMP1 as biomarker for PCa, unveiling its biological relevance in prostate carcinogenesis. Microarray analyses of treated PCa cell lines and primary tissues enabled the selection of differentially methylated genes, among which *EFEMP1* was further validated by MSP and bisulfite sequencing. Assessment of biomarker performance was accomplished by qMSP. Expression analysis of *EFEMP1* and characterization of histone marks were performed in tissue samples and cancer cell lines to determine the impact of epigenetic mechanisms on *EFEMP1* transcriptional regulation. Phenotypic assays, using transfected cell lines, permitted the evaluation of *EFEMP1*’s role in PCa development. *EFEMP1* methylation assay discriminated PCa from normal prostate tissue (NPT; *P* < 0.001, Kruskall–Wallis test) and renal and bladder cancers (96% sensitivity and 98% specificity). *EFEMP1 t*ranscription levels inversely correlated with promoter methylation and histone deacetylation, suggesting that both epigenetic mechanisms are involved in gene regulation. Phenotypic assays showed that *EFEMP1 de novo* expression reduces malignant phenotype of PCa cells. *EFEMP1* promoter methylation is prevalent in PCa and accurately discriminates PCa from non-cancerous prostate tissues and other urological neoplasms. This epigenetic alteration occurs early in prostate carcinogenesis and, in association with histone deacetylation, progressively leads to gene down-regulation, fostering cell proliferation, invasion and evasion of apoptosis.

## Introduction

Prostate cancer (PCa) is one of the major public health issues, standing as the second most incident cancer in the male population worldwide (only surpassed by lung cancer) and the fifth most common cancer overall 1. Although screening for PCa by PSA testing combined with digital rectal examination has been established in some countries, the evidence of the benefit of this screening is controversial 2. However, these tools are well accepted and widely used as ancillary tools for earlier detection of PCa. The issue of early detection is critical in PCa because only organ-confined disease is amenable to curative treatment, whereas patients with advanced disease can only be palliated. Regardless of the utility of screening tools, the only available diagnostic approach for this malignancy is the histopathological analysis of prostatic tissue obtained from biopsy 3. Thus, the development of new diagnostic tools would be a great achievement for patients undergoing PCa screening.

Carcinogenesis is characterized by the accumulation of both genetic and epigenetic alterations. Remarkably, the number of genes involved in the development and progression of tumour which are epigenetically silenced probably surpasses the number of genes inactivated by mutation 4. Therefore, epigenetic alterations might be used as disease biomarkers, providing important information for early detection, diagnosis and prognosis of malignant diseases that are usually asymptomatic at early stages 5. DNA methylation is critical for the regulation of multiple cellular events and it is by far the most studied epigenetic mechanism in cancer. Alterations of methylation patterns have been proved to be implicated in carcinogenesis and DNA hypermethylation of several genes has been observed in a wide range of different tumour types 6,7 and suggested as potential cancer biomarkers 8. Strikingly, over the last decade, the interplay between different epigenetic mechanisms such as DNA methylation and histone post-translational modifications (PTMs) on gene transcription 9 as well as its role in tumourigenesis has become evident. This has been considered an important step towards the understanding of the biological relevance of epigenetic alterations in cancer, apart from their potential usefulness in diagnosis.

In the search for methylation-based biomarkers intended for PCa detection and assessment of clinical aggressiveness, we have used a combined strategy of epigenetic drug treatment of PCa cell lines followed by microarray-based expression analyses, and compared the re-expressed genes with those that are down-regulated in primary tumours 10. This genome-wide strategy allowed for the identification of the EGF containing fibulin-like extracellular matrix protein 1 (*EFEMP1*) gene as a novel potentially epigenetically deregulated gene in PCa. Herein, we sought to validate *EFEMP1* promoter methylation as a potential PCa biomarker as well as to explore the role of DNA methylation and histone PTMs in *EFEMP1* down-regulation (transcript and respective protein – Fibulin-3 – levels) and its impact in on PCa tumourigenesis, thus uncovering the biological relevance of *EFEMP1* epigenetic deregulation.

## Cell and tissue samples

### Prostate cell lines

Cell lines representing PCa (22Rv1, DU145, LNCaP, PC-3, VCaP) and non-neoplastic prostatic epithelium (PNT2) were grown in appropriate media, as detailed elsewhere 11,12.

### Patients and tissue sample collection

Prostate cancer samples were prospectively collected from patients with clinically localized disease, consecutively diagnosed and treated with radical prostatectomy at the Portuguese Oncology Institute – Porto, Portugal. For control purposes, 32 benign prostate hyperplasia (BPH) and 15 normal prostate tissues (NPT; non-cancerous prostate tissues) were used. BPH and NPT samples were collected from patients who underwent transurethral resection of the prostate and from cystoprostatectomy specimens of bladder cancer (BCa) patients respectively.

Two main series of PCa tissues were available for the purposes of this study: a test group of 24 samples selected from a pool of 112 patients (used for expression array analysis), and a validation group comprising 201 consecutive samples obtained in the same cancer centre. Paired high-grade PIN (HGPIN) lesions were also identified in 73 of these 201 specimens and collected for further analysis.

Furthermore, tissue samples from 25 BCa and 73 renal cell tumour (RCT) [comprising 18 clear cell renal cell carcinoma (ccRCC), 16 papillary RCC (pRCC), 19 chromophobe RCC (chRCC) and 20 oncocytomas (Onc)] were also collected from patients diagnosed and treated at the Portuguese Oncology Institute – Porto, Portugal. All tissue specimens were promptly frozen immediately after surgery and stored at −80°C for further analysis.

Histological slides from formalin-fixed, paraffin-embedded tissue fragments were routinely obtained from the same surgical specimens, and histopathologically assessed, including Gleason grading 13 and staging 14.

Relevant clinical data were collected from the clinical charts. All patients were enrolled in this study after informed consent. These studies were approved by the institutional review board [Comissão de Ética para a Saúde-(IRB-CES-IPOFG-EPE 019/08)] of Portuguese Oncology Institute – Porto, Portugal.

### Isolation of nucleic acids

DNA from all samples was extracted by phenol–chloroform conventional method. Total RNA was extracted from cell lines pellets using TRIzol® Reagent (Invitrogen) according to the manufacturer’s manual. RNA extraction from the first 100 PCa samples of the validation series and all the 15 NPT samples was carried out using PureLink™ RNA Mini Kit (Invitrogen). DNA and RNA samples were stored at −20°C and −80°C respectively.

## Gene selection and validation

### Gene expression microarrays

Four PCa cell lines (22Rv1, DU145, LNCaP and PC-3), both untreated and treated with a combination of the demethylating drug 5-aza-2′deoxycytidine (DAC, 1 μmol/l for 72 hrs) and the histone deacetylase inhibitor trichostatin A (TSA, 0.5 μmol/l added in the last 12 hrs), were analysed with the Applied Biosystems Human Genome Survey Microarray (P/N 4337467), as extensively described in 15.

Microarray analysis of PCa (*n* = 24) and NPT samples (*n* = 6) was carried out in parallel. The relative gene expression in tumour samples was calculated using the median value of expression of the normal tissues. Arrays elements, up-regulated more than fourfold after DAC and TSA treatment in at least 2 of 4 PCa cell lines and simultaneously down-regulated in tumour samples compared with non-malignant tissue, were considered to be potential targets for epigenetic regulation.

### *In silico* screening for CpG islands, DNA bisulfite modification, methylation-specific polymerase chain reaction (MSP) and bisulfite sequencing

Potential candidate genes for down-regulation by promoter methylation were analysed for the existence of a CpG island in their promoter region, as described elsewhere 10,15, but now including the 2000-bp sequence upstream of the first exon. A selected list of candidate genes screened by MSP is provided in Table S1.

Genes with a CpG island in the promoter region were then screened by Methylation-Specific Polymerase Chain Reaction (MSP) in bisulfite-modified DNA from the four cell lines analysed in the microarray. For this purpose, cytosine-rich regions of their promoters were selected for primer design using Methyl Primer Express Software v1.0 (Applied Biosystems). When appropriate, previously published primers pairs, including for *EFEMP1*, were also tested (data not shown) 16.

Genomic DNA (1 μg) from cell lines was subjected to a chemical modification by sodium bisulfite using EZ DNA Methylation-Gold™ Kit (Zymo Research) according to manufacturer’s protocol. All MSP reactions were performed according to DyNAzyme™ II Hot Start manufacturer’s conditions. Bisulfite-modified CpGenome™ Universal Methylated DNA (Millipore) and donor lymphocyte DNA were included in each PCR to serve as positive and negative controls respectively. All PCR products were loaded onto 2% agarose gels, stained with ethidium bromide and visualized under an ultraviolet transilluminator.

To confirm MSP results and to obtain detailed information about the methylation status of CpG sites, bisulfite sequencing PCR (BSP) was performed in one normal immortalized prostatic cell line – PNT2 – and five PCa cell lines – 22Rv1, DU145, LNCaP, PC-3 and VCaP for *EFEMP1*, using primers that span the region of interest 17. PCR reactions were performed as previously described 10,15. *EFEMP1* was then selected for further analysis because it was methylated all PCa cell lines analysed.

### Quantitative methylation-specific polymerase chain reaction (qMSP)

Methylation levels analysis by qMSP was performed as previously described 10. In brief, bisulfite-modified DNA from the validation set of clinical samples (201 PCa, 73 HGPIN, 15 NPT and 32 BPH) was tested using qMSP to verify the methylation status of this gene promoter and its potential as a PCa biomarker. To confirm its tumour specificity, *EFEMP1* promoter methylation was also evaluated in other common urological neoplasms (BCa and RCT).

*EFEMP1* primers for this assay were designed according to bisulfite sequencing results to encompass those CpG dinucleotides that were methylated in the majority of the five PCa cell lines analysed. All samples were also tested using primers for an internal reference gene (*ACT*β) to normalize results for the bisulfite-modified DNA input of each sample. Primers and probes are displayed in Table S2.

Quantitative MSP was performed in a 7000 Sequence Detection System (Applied Biosystems) and all samples were run in triplicate. The standard curve method was used for quantification purposes. PCR conditions were optimized for each pair of primers using AmpliTaq® Gold DNA Polymerase (5 U/μl; Applied Biosystems) according to manufacturer’s protocol. The results were analysed using Sequence Detection Software version 1.2.3 (Applied Biosystems).

The relative level of *EFEMP1* methylation in each sample was determined using the following formula 18: [(*EFEMP1* sample/*EFEMP1* universal methylated DNA)/(*ACT*β sample/*ACT*β universal methylated DNA)]. The ratio was then multiplied by 1000 for easier tabulation.

## Assessment of *EFEMP1* regulation by epigenetic mechanisms in PCa

### Quantitative gene expression analysis

Gene expression assays were performed and analysed as previously described 19. Briefly, RNA extracted from the first 100 PCa samples of the validation series and from all NPT samples was reversely transcribed and amplified using the TransPlex Whole Transcriptome Amplification (WTA) Kit (Sigma-Aldrich), according to manufacturer’s instructions. All WTA-cDNA samples were 5× diluted, and *EFEMP1* gene expression assay (Hs00244575_m1 from Applied Biosystems) and the endogenous control assay *GUSB* (Hs99999908_m1 from Applied Biosystems) were used to quantify gene expression by Real-Time PCR using a 7000 Sequence Detection System (Applied Biosystems) according to recommended protocol. All samples were run in triplicate. The standard curve method was used for quantification purposes [(EFEMP1/BGUS)×1000]. Results were analysed using the Sequence Detector Software version 1.2.3 (Applied Biosystems).

### Cell line treatment with epigenetic modulating drugs

To confirm whether *EFEMP1* expression was regulated by promoter methylation and/or histone PTMs, three PCa cell lines (LNCaP, PC-3, VCaP) were treated with 1 μM DAC for 72 hrs, 0.5 μM TSA for 24 hrs or both in combination. All the treatments were performed in triplicate.

From each sample, RNA was extracted and reversed transcribed into cDNA using the RevertAidTM H *Minus* First Strand cDNA Synthesis Kit (Fermentas), according to the manufacturer’s indications. Gene expression assays were performed and analysed as previously described 15.

Methylation levels analysis by qMSP was also performed, as previously described, using bisulfite-modified DNA extracted from both treated and untreated cell lines 15.

### Chromatin immunoprecipitation assay

EZ-Magna chromatin immunoprecipitation (ChIP) G-Chromatin Immunoprecipitation Kit and the Magna Grip Rack (Millipore) were used to perform ChIP assay in LNCaP cells according to manufacturer’s instructions. Antibodies against histone H3 (ab1791; Abcam), histone H4 (ab70701; Abcam), AcH3 (06-599; Upstate), AcH4 (06-866; Upstate), H3K4me3 (ab8580; Abcam) and H3K9ac (CS200583; Millipore) were used.

Primers were designed for three different regions upstream to transcriptional start site (Table S2). Firstly, the relative amount of promoter DNA was normalized using input percent method. The enrichment over the core histone (H3 or H4) was then calculated for the appropriate histone in each case.

## Evaluation of the biological relevance of Fibulin-3 in prostate carcinogenesis

### Transient transfection

To uncover the biological role of fibulin-3, the protein encoded by *EFEMP1*, in PCa, PC-3 and LNCaP cells were transiently transfected with transfection-ready DNA containing *EFEMP1* transcript variant 1 (Origene Technologies), following the manufacturer’s instructions, for 48 h (the time-point found to correspond to the highest *EFEMP1* expression). For control purposes, an empty vector was also transfected to PC3 and LNCaP cells. cDNA was reversely transcribed from RNA using the High Capacity cDNA Reverse Transcription Kit (Applied Biosystems). *EFEMP1* gene expression analysis was performed as previously described 19.

### Protein extraction and western blot

Forty-eight hours after transfection, whole-cell protein extraction and western blot were performed as described elsewhere 12. Membranes were probed with antibodies against Fibulin-3 (SantaCruz Biotechnology, at 1:500) or the endogenous control β-actin (Sigma-Aldrich, A1978, at 1:10,000). All experiments were performed in triplicates.

### Cell viability assay

Cells were seeded in 96-well plates under standard conditions until acquisition of a 30% to 50% confluence. Transient transfection was then performed, cell cultures were maintained for 48 hrs and subsequently cell viability was measured by the 3-(4,5-dimethylthiazol-2-yl)-2,5-diphenyltetrazolium-bromide (MTT) assay, as described in 12. Three independent experiments were performed, using triplicates for each experiment.

### Apoptosis Assay

PC-3 and LNCaP cells were prepared as for MTT assay and cell apoptosis was quantified using the APOPercentage apoptosis assay kit (Biocolor Ltd), according to the manufacturer’s instructions and as described in 12. Three independent experiments were performed, using nine replicates for each experiment.

### Invasion assay

Cell invasion in PC-3 after *EFEMP1* overexpression was evaluated using the BD BioCoat Matrigel Invasion Chamber (BD Biosciences), according to manufacturer’s indications. For quantification, cells were counted under a fluorescent microscope through all membrane and the assay was performed in triplicates. Data are expressed as the percentage of invasive cells through the membrane, in comparison to control cells.

### Statistical analysis

For each group of tissue samples, median and interquartile (P25–P75) range of *EFEMP1* methylation levels were determined, and were compared using Kruskall–Wallis or Mann–Whitney *U*-test, depending on the number of categories in each group. Comparison of methylation levels of paired PCa and HGPIN samples was carried out using the Wilcoxon Signed Rank test. The relationship between methylation ratios and standard clinicopathological variables (tumour grade and stage) was evaluated using the same tests. A Spearman non-parametric correlation test was performed to correlate age and serum PSA levels at diagnosis with *EFEMP1* methylation levels. Frequency was also calculated using as a cut-off the higher methylation level of NPT samples. To assess the diagnostic performance of *EFEMP1* quantitative promoter methylation, receiver operator characteristics (ROC) curves were constructed by plotting the true positive rate (sensitivity) against the false-positive rate (1-specificity), and the area under the curve (AUC) was calculated. The correlation between *EFEMP1* promoter methylation and transcript levels in PCa and NPT tissue samples was assessed using Spearman non-parametric test.

For cell lines, differences in transcript and methylation levels among tested treatments were determined using one-way anova test, followed by a multiple comparisons Dunnet’s test, comparing all groups against the Mock.

In cell lines, differences in transcript after transfection and in phenotypic features after *EFEMP1* overexpression were determined using a Student’s *t*-test, comparing all groups against the untreated control.

All tests were two-sided and *P*-values were considered significant when inferior to 0.05. For multiple comparisons the Bonferroni’s correction was applied. Statistical analyses were performed with a computer-assisted program (SPSS version 20.0, USA).

## Results

### *EFEMP1* promoter methylation accurately discriminates PCa from non-cancerous prostate tissues and urological tumours

**Figure 1:**
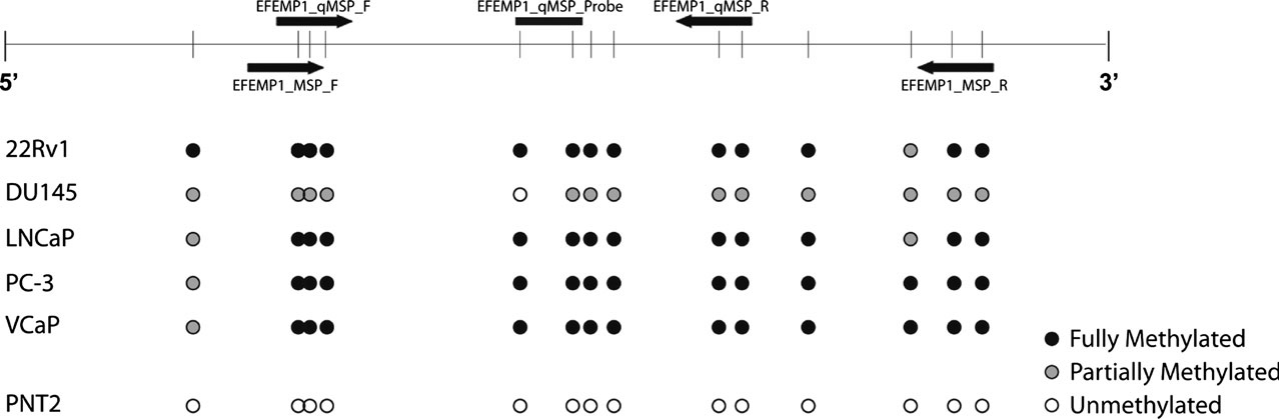
Assessment of *EFEMP1* promoter methylation status in prostate cell lines of individual CpG dinucleotides by bisulfite sequencing. The upper panel diagram represents the region of the gene under analysis, MSP and quantitative MSP (qMSP) primers and probe localization (black arrows and horizontal bar respectively) and CpG dinucleotides density (vertical bars). The lower panel shows the status of methylation for each CpG dinucleotide for five different prostate cancer cell lines (22Rv1, DU145, LNCaP, PC-3, VCaP) and an immortalized normal prostate cell line (PNT2). White circle – unmethylated CpG; grey circle – partially methylated CpG; black circle – fully methylated CpG.

**Figure 2:**
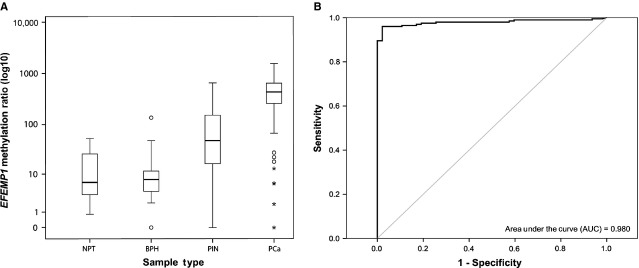
Distribution of *EFEMP1* promoter methylation levels in prostate tissue samples and its performance as a cancer biomarker. (**A**) Methylation levels of *EFEMP1* promoter region, using qMSP, in benign prostate hyperplasia (BPH), normal prostate (NPT), high-grade prostatic intraepithelial neoplasia (HGPIN) and prostate adenocarcinoma (PCa). (**B**) ROC (receiver operating characteristic) curve evaluating the performance of *EFEMP1* methylation levels as a biomarker to discriminate between malignant and non-malignant prostatic tissues (AUC, area under the curve; CI, confidence interval). Statistically significant differences between the Mock and treatment groups are denoted as: *P < 0.05; **P < 0.01; ***P < 0.001.

*EFEMP1* was found to be methylated in all PCa cell lines analysed (Fig. 1) and it was then selected for further analysis. *EFEMP1* promoter methylation was detected in most primary PCa (96%), but only in a minority of non-cancerous prostate tissues (3% and 7%, for BPH and NPT respectively), as well as in 51% of HGPIN lesions. Methylation levels, assessed by qMSP, differed significantly among prostate tissue samples (*P* < 0.001, Kruskall–Wallis test). PCa samples showed significantly higher *EFEMP1* methylation levels compared with HGPIN, BPH and NPT (*P* < 0.001, Mann–Whitney *U*-test; Fig. 2A and Table S3). HGPIN lesions displayed *EFEMP1* methylation levels intermediate between PCa, on the one hand, and BPH and NPT, in the other, and differences were of statistical significance (*P* < 0.001 for all comparisons, Mann–Whitney *U*-test). HGPIN samples paired with tumours displayed significantly lower levels of methylation (*P* < 0.001 for Wilcoxon Signed Rank test).

The relevant clinicopathological features of the patient’s populations are depicted in Table S4. No statistically significant association was found between *EFEMP1* methylation levels and any of the clinicopathological variables of PCa patients.

To assess the biomarker performance of *EFEMP1* quantitative promoter methylation, the higher methylation level determined in non-malignant tissue samples was used as empirical cut-off (53.2). Validity estimates for sensitivity, specificity, positive and negative predictive value, and accuracy are provided in Table S5. *EFEMP1* methylation levels distinguished PCa from NPT with 96.02% sensitivity and 97.87% specificity. ROC curve analysis displayed an AUC of 0.980 [95% confidence interval (CI): 0.963–0.996; *P* < 0.001] (Fig. 2B). Remarkably, no *EFEMP1* promoter methylation was found in the vast majority of tested BCa and RCT samples, emphasizing the tumour specificity of this methylation assay (Fig. S1A and Table S6). ROC curve analysis confirmed that *EFEMP1* methylation levels were able to discriminate PCa from the other urological tumours with high sensitivity and specificity (AUC = 0.986; 95% CI: 0.974–0.998; *P* < 0.001; Fig. S1B and Table S7).

### Transcriptional repression of *EFEMP1* is because of epigenetic alterations in PCa

**Figure 3:**
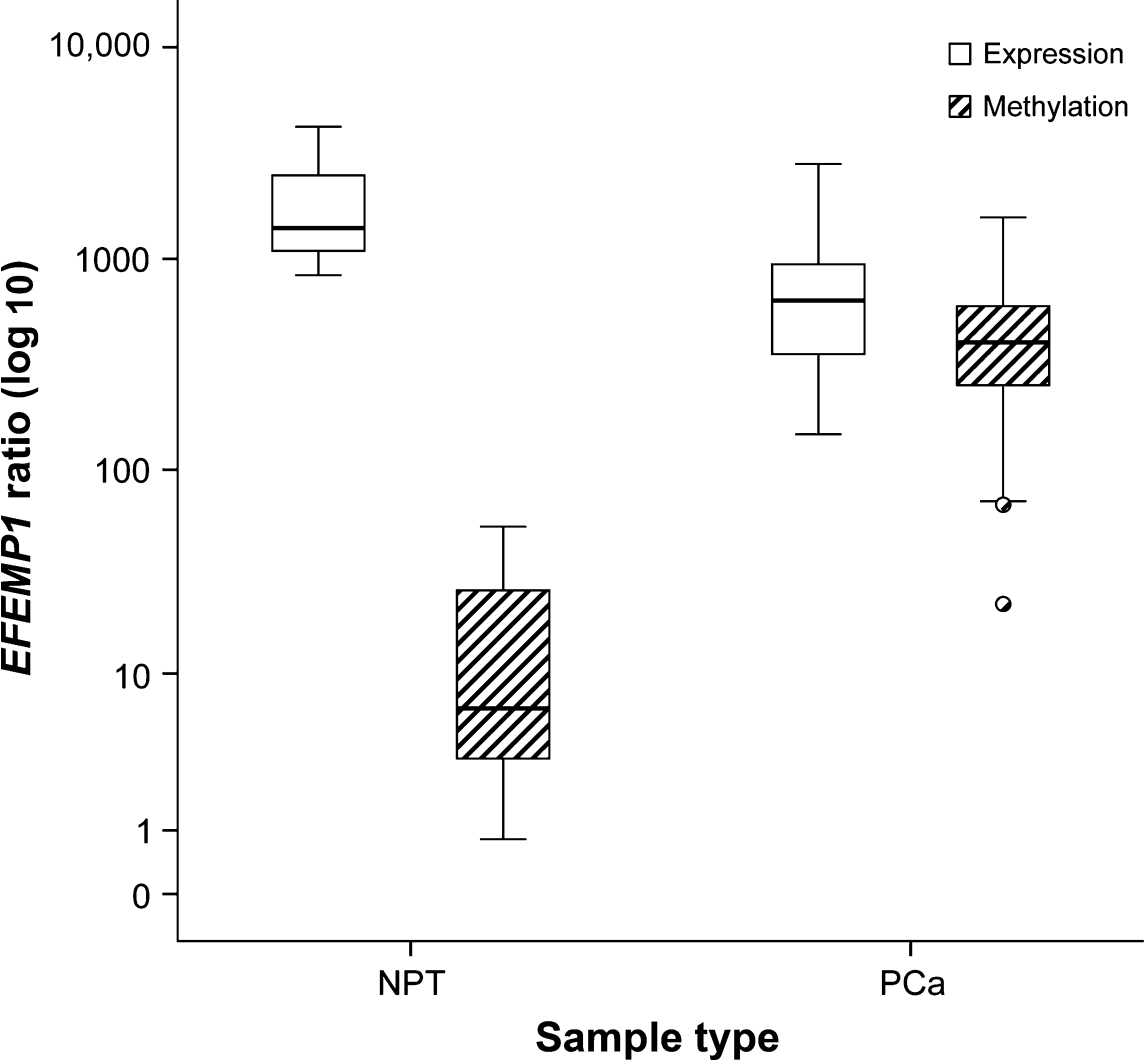
*EFEMP1* promoter methylation and mRNA expression levels (assessed by real-time PCR) in normal (NPT) and cancerous (PCa) prostate tissue samples. Lower transcript levels of *EFEMP1* were found in PCa tissues compared with NPT (*P* < 0.001, Mann–Whitney *U*-test), which inversely correlated with promoter methylation levels (*r* = −0.403; *P* < 0.001).

Significantly lower transcript levels of *EFEMP1* were found in PCa tissues compared with NPT (*P* < 0.001, Mann–Whitney *U*-test) and those inversely correlated with promoter methylation levels (*r* = −0.403; *P* < 0.001) (Fig. 3), suggesting an association between *EFEMP1* promoter methylation and gene silencing in PCa.

**Figure 4:**
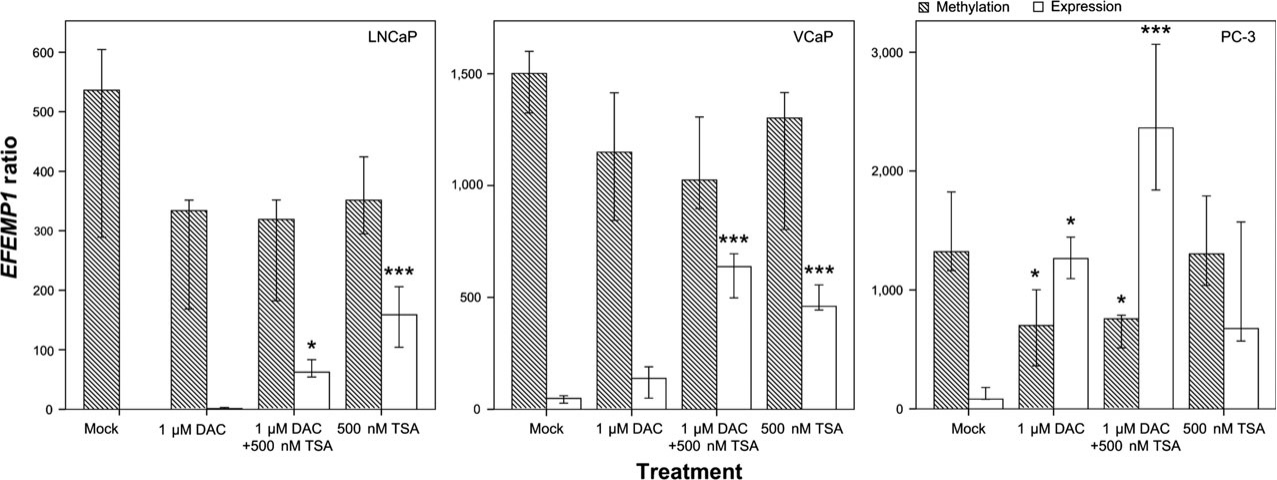
*EFEMP1* promoter methylation and mRNA expression levels in prostate cancer cell lines (LNCaP, VCaP, PC-3) after pharmacological treatment with 5-aza-2′deoxycytidine alone (1 μM DAC), in combination with trichostatin A (1 μM DAC+ 500 nM TSA) or with trichostatin A alone (500 nM TSA). Error bars represent mean ± SD of 3 biological replicates. Statistically significant differences between the Mock and treatment groups are denoted as: *P < 0.05; **P < 0.01; ***P < 0.001.

To further explore the role of epigenetic mechanisms, namely DNA methylation and histone acetylation, in *EFEMP1* transcriptional repression, three PCa cell lines, representative of different androgen-sensitivity status and ETS rearrangements, were treated with DAC and/or TSA. *EFEMP1* DNA methylation and transcript levels were quantitatively assessed (Fig. 4). All cell lines demonstrated low or absent levels of *EFEMP1* transcript concomitant with high methylation levels of the respective promoter. Following exposure to epigenetic modulating drugs, LNCaP and VCaP cell lines showed a slight re-expression upon treatment with demethylating agent DAC, concurrently with a decrease, although non-significant, in methylation levels. However, a significant increase in mRNA levels was depicted in both cell lines following exposure to TSA alone or in combination with DAC (*P* = 0.019 for combined treatment in LNCaP and *P* < 0.001 for the remainder), although without a significant variation in *EFEMP1* promoter methylation levels. Concerning PC-3 cells, a significant decrease in methylation levels was apparent upon exposure to DAC alone or in combination with TSA (*P* = 0.023 for both treatments), with a concomitant increase in gene expression levels (*P* = 0.013 for DAC alone and *P* < 0.001 for combined treatment). Indeed, a significant inverse correlation between *EFEMP1* methylation and transcript levels was detected [*r* = −1, *P* < 0.01, Spearman’s test]. Strikingly, increase in *EFEMP1* expression was more impressive after combined treatment.

**Figure 5:**
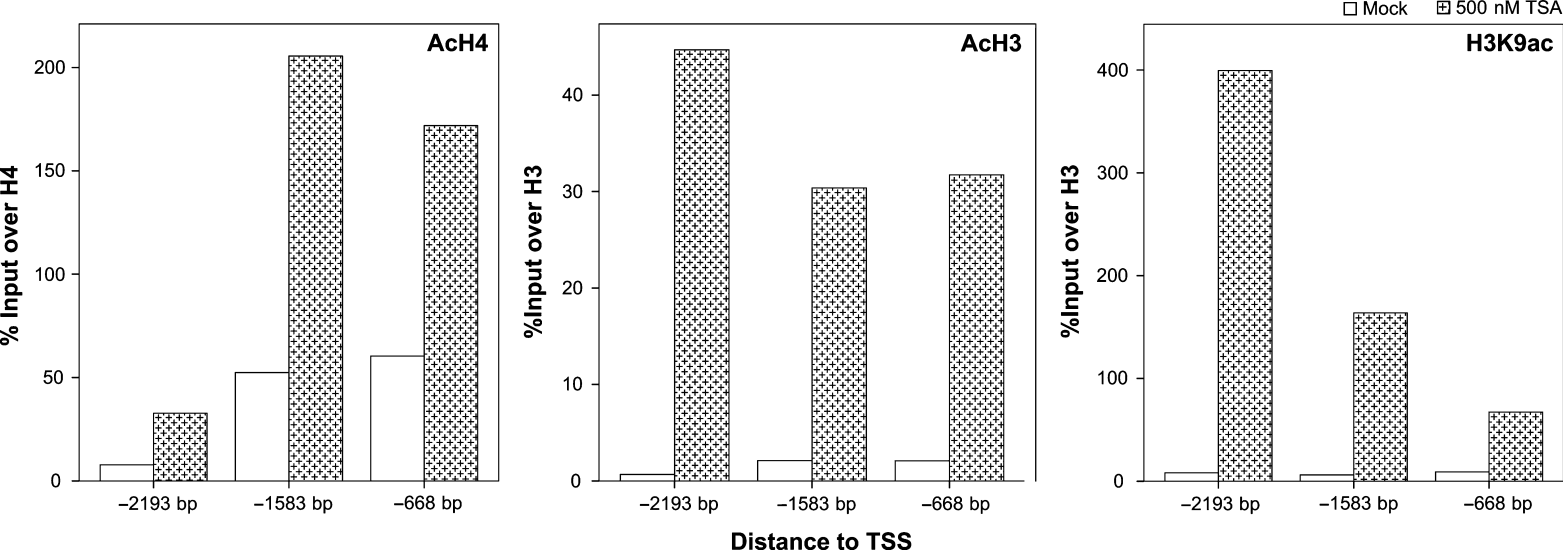
Quantitative ChIP-PCR analysis of post-translational histone modifications (PTMs) associated with transcriptional activation (AcH4, AcH3, H3K9ac). There is an enrichment in three different regions upstream of *EFEMP1* transcriptional start site (TSS) in LNCaP cell line upon treatment with trichostatin A (TSA, 500 nM). Positive and negative ChIP-controls (RNA polymerase 2 and IgG respectively) are not depicted. Results are represented as percentage of input over control (H4 or H3, accordingly).

Based on these results, we hypothesized that histone acetylation status might be involved in regulation of *EFEMP1* transcription. Thus, we performed ChIP to ascertain the global acetylation status of Histones H3 and H4 and acetylation of H3K9 using LNCaP and PC-3 cell lines (Fig. 5). Interestingly, histone acetylation was increased in the three regions of *EFEMP1* upstream to TSS upon treatment of PCa cell lines with TSA, although with different distribution of the activating marks along the promoter. A more impressive increase was apparent for H3K9ac and AcH4 closer to the TSS, whereas AcH3 was more likely to accumulate at distance from the TSS, particularly for PC-3 cells.

### *EFEMP1 de novo* expression impairs the malignant phenotype of PCa cells *in vitro*

**Figure 6:**
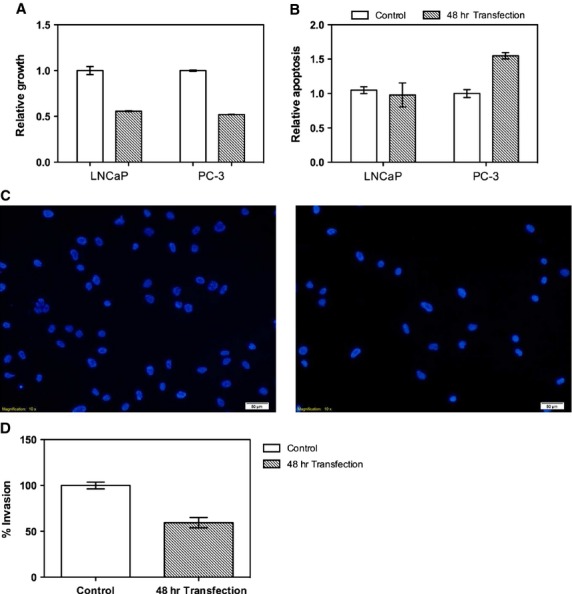
Impact of *EFEMP1 de novo* expression on the malignant behaviour of PCa cell lines (**A**) After 48 hrs of transfection, cell viability significantly decreased in LNCaP and PC-3 cell lines; (**B**) *EFEMP1* transfection increased apoptosis levels in PC-3, but not LNCaP cells; (**C**) *EFEMP1* transfection decreased invasive potential of PC-3 cells (right) compared with control (empty-vector) (left). (**D**) Percentage of invasion determined as recommended by manufacturer. Data are shown as relative growth, relative apoptosis and percentage of invasion in comparison to empty-vector transfected cells (mean ± SD, *n* = 3). Error bars represent mean ± SD of 3 biological replicates. Statistically significant differences are denoted as: ***P < 0.001, ****P < 0.0001.

Transient transfection of *EFEMP1* was carried out to increase *EFEMP1* expression in LNCaP and PC3 cells and its effect on the malignant phenotype was evaluated. Both transcript and protein levels of *EFEMP1* were significantly increased after transfection (Fig. S2). *De novo* expression of Fibulin-3 induced a decrease in cell viability of LNCaP and PC-3 cells (*P* < 0.0001, for both) (Fig. 6A), whereas a significant increase in apoptosis was only observed in PC3 (*P* < 0.001) (Fig. 6B). Invasion assay was performed in PC-3 cell line because its invasive capacity has been largely documented and it showed the most impressive phenotypic alterations after *EFEMP1* transient transfection. Remarkably, Fibulin-3 forced expression significantly decreased the invasive potential of PC-3 (Fig. 6C) compared with empty-vector containing cells (*P* < 0.001) (Fig. 6D).

## Discussion

Prostate cancer is a major health issue worldwide, and especially in western countries 3. Notwithstanding the controversies regarding the use of serum PSA for PCa screening, which displays a modest performance (21% sensitivity and 91% specificity) 20, pathological evaluation of prostate biopsies remains the gold standard diagnostic tool, and the development of less invasive and more cost-effective diagnostic methods would be a major achievement 21. Moreover, a conservative approach in the evaluation of core biopsy specimens among pathologists entailed the development of more accurate ancillary tools to perfect PCa diagnosis and assess disease aggressiveness 22. Thus, gene-specific DNA methylation has been evaluated as a potential diagnostic tool, holding promise for clinical implementation in the short term 23. Using a genome-wide search for genes down-regulated as a result of epigenetic alterations, we identified *EFEMP1* promoter methylation as a promising PCa biomarker. To substantiate this finding, we found that promoter methylation levels increase from non-cancerous prostate tissues to HGPIN to PCa, inversely correlating with transcript levels, thus, indicating a role for *EFEMP1* expression perturbation in prostate carcinogenesis. Finally, *in vitro* assays demonstrated that *de novo* expression of Fibulin-3 attenuates the malignant phenotype of PCa cells.

Our findings concerning the biomarker performance of *EFEMP1* quantitative promoter methylation for PCa detection parallel those of a previously published study 24, reinforcing the potential clinical interest of these results. Although the strategy that led to the identification of *EFEMP1* methylation is similar in both studies (which serves as reciprocal methodological validation), important differences should be emphasized. Our study is the first to assess *EFEMP1* promoter methylation in PCa tissues from a western population, as that of Kim and coworkers included only Korean men 24. This is a relevant issue as relevant ethnic differences in gene methylation profile of PCa have been reported 25. Furthermore, our series almost doubles that of Kim *et al*. and included NPT from the peripheral zone as controls, which might justify the better specificity found in our study (97.87% *versus* 86.6% 24), although sensitivity was almost the same. In addition, we also tested, for the first time, the tumour site-specificity of *EFEMP1* methylation, demonstrating that it is PCa-specific. This finding supports the use of *EFEMP1* methylation for PCa detection in bodily fluids such as urine or serum/plasma (and which has not been tested in any of the studies) allowing for the discrimination from BCa and RCT, that were shown by us and others to be diagnosable in urine samples 15,26,27. Moreover, our series also included HGPIN tissue samples, which are considered the most likely precursor lesions of PCa. The analysis of these samples enabled us to verify that *EFEMP1* promoter methylation is acquired during the early phases of prostate carcinogenesis and progressively increases as the full-blown malignant phenotype is established. Finally, we performed phenotypic assays that sustain an important biological role of Fibulin-3 in PCa cells. We thus believe that the present study not only confirms but also adds novel information concerning *EFEMP1* promoter methylation in PCa.

The high accuracy (96%) of *EFEMP1* methylation assay in tissue samples shows promise for its use as an ancillary tool for diagnostically challenging lesions of the prostate, especially in biopsy specimens, emphasizing the need to combine molecular tests and routine histopathological examination. Compared with other commonly methylated genes in PCa (*e.g. GSTP1*, *APC* and *RAR*β) 28, the *EFEMP1* methylation assay demonstrates an equivalent performance, even when compared with multigene testing 29. Indeed, considering all tumour and non-cancerous tissue samples tested, only 10 out of 356 would be misclassified according to the *EFEMP1* methylation test: eight PCa would not be detected and only 1 BCa and 1 RCT would be misdiagnosed as PCa. Clearly, further studies are required to validate our results and determine the feasibility of assessing *EFEMP1* methylation in urine samples, enabling the development of a clinically useful non-invasive method.

Interestingly, no associations were found between *EFEMP1* methylation levels and standard clinicopathological parameters, underscoring its potential as a biomarker for detecting early stages of PCa. Moreover, as previously stated, the prevalence of high and intermediate methylation levels of *EFEMP1* in PCa and HGPIN samples, respectively, suggest that *EFEMP1* methylation is an early event in prostatic carcinogenesis, further supporting its use as a biomarker for PCa detection. Additionally, the fact that this gene was reported to be silenced by promoter methylation in other cancer types, namely those of the breast 17, lung 16 and endometrium 30, indicates that this event is likely to be relevant in carcinogenesis. However, this is likely to be a tumour-type specific feature, because Fibulin-3, the protein encoded by *EFEMP1*, was reported to be overexpressed in glioma 31, cervical cancer 32, and pleural mesothelioma 33.

Fibulin-3 is a member of the fibulins family, which are extracellular matrix (ECM) glycoproteins associated with basement membranes, elastic fibres and matricial components 34,35. Three members of this family (Fibulin-1, Fibulin-4 and Fibulin-5) were previously reported to be down-regulated in PCa 35. Here, we demonstrated that *EFEMP1* mRNA levels are reduced in PCa compared with NPT, confirming previous findings 24. Remarkably, we verified that PCa cell lines displaying reduced transcript levels also show reduction of Fibulin-3 at the protein level, emphasizing the role of this family of proteins in prostate carcinogenesis. Importantly, promoter methylation levels inversely correlated with expression levels both in PCa tissues and cell lines, suggesting that promoter methylation plays a major role in *EFEMP1* silencing. Nevertheless, our attempt to revert gene silencing in PCa cell lines using epigenetic modulating drugs showed that this mechanism may not be acting alone in gene transcription regulation, unveiling a potential role for histone PTMs, particularly histone acetylation. Indeed, ChIP results demonstrated an enrichment of activating histone marks (AcH4, AcH3, H3K9ac) upstream of TSS upon TSA treatment in LNCaP and PC-3 cell lines. Interestingly, LNCaP, VCaP and PC-3 cells responded differently to treatment with DAC, TSA alone or combined, potentially reflecting different biological subgroups within prostate adenocarcinomas. Remarkably, cell lines sharing the same androgen-sensitivity status (LNCaP and VCaP *versus* PC-3) displayed similar results, a finding that might be related to the putative regulation of *EFEMP1* expression by androgens 36,37.

PC-3 (a castration-resistant cell line) showed higher *EFEMP1* re-expression upon DAC treatment compared with the other two PCa cell lines (although it showed the higher methylation levels), suggesting that DNA methylation might be more relevant for ‘locking’ gene silencing in more clinically advanced PCa, whereas histone PTMs, a more dynamic transcription regulator, might be more relevant in the early stage, androgen-responsive, tumours. In this setting, it is tempting to speculate whether *EFEMP1* methylation levels might predict tumours more prone to progress to an androgen-independent status. This issue could not be assessed in our series of patients as all were clinically localized PCa, not previously exposed to androgen-deprivation therapy. Regardless of a potential hormonal regulation of the gene, it was clearly demonstrated that *EFEMP1* is epigenetically deregulated in PCa, and that a dynamic interplay between histone PTMs and DNA methylation takes place during tumourigenesis resulting in effective gene silencing 36,37.

In normal tissues, Fibulin-3 is usually highly expressed by epithelial and endothelial cells, interacting with several other proteins of the ECM, not only contributing to the integrity of the basement membrane but also to the assembly of elastic fibres during embryonic development 34,35. Moreover, Fibulin-3 inhibits the activity of several matrix metalloproteinases, thus participating in the stable organization of ECM structures as well as in the reduction of its proteolysis and remodelling 34,38. Remarkably, cell lines transfected with *EFEMP1* express high levels of E-cadherin and low levels of vimentin 30. In addition to its structural importance, Fibulin-3 has signalling properties as its expression is inversely correlated with cell growth 34. Because Fibulin-3 is important to maintain cellular and tissue homeostasis, its deregulation might lead to deregulated cell growth, invasion and modification of ECM, which are hallmarks of cancer cells 38. Furthermore, morphological analysis of PCa tissues revealed a total lack of basal cell layer and basal membrane, which correlates well with deregulation of ECM proteins, including Fibulin-3 39. Interestingly, the phenotypic assays showed that *EFEMP1 de novo* expression in PCa cell lines impacted mainly on tumour cell viability. Our results (which are in line with those reported for endometrial cancer cells) 30 also suggest that, at least in some PCa, *EFEMP1* silencing promotes the emergence of an invasive phenotype as well as resistance to apoptosis. The attenuation of these features upon gene *in vitro* re-expression highlights the biological relevance of *EFEMP1* epigenetically mediated down-regulation in PCa. Thus, we hypothesize that *EFEMP1* promoter methylation might be a driver epimutation in prostate carcinogenesis.

We concluded that *EFEMP1* promoter methylation is a prevalent feature of PCa, accurately discriminating PCa from non-cancerous prostate tissues and other urological neoplasms. This epigenetic alteration occurs early in prostate carcinogenesis and, in association with histone deacetylation, progressively leads to gene down-regulation, fostering cell proliferation, invasion and evasion of apoptosis.

## Supporting Information

Figure S1 Distribution of *EFEMP1* methylation levels in urological tumour samples and their performance as a prostate cancer biomarker.

Figure S2 Validation of *EFEMP1* transfection.

Table S1 Genes selected by microarray gene expression analysis as potential epigenetic biomarkers that were screened by MSP in this study. **Table S2** Primers designed for qMSP and for ChIP assay. **Table S3** Distribution of *EFEMP1* methylation levels quantified by qMSP and methylation frequency in prostatic tissues. **Table S4** Clinical and histopathological features of the patients and controls. **Table S5** Diagnostic performance parameters of *EFEMP1* methylation levels to distinguish NMT from PCa. **Table S6** Distribution of *EFEMP1* methylation levels quantified by qMSP and methylation frequency in other urological tumours. **Table S7** Diagnostic performance parameters of *EFEMP1* methylation levels to distinguish PCa from other urological tumours (BCa, RCT).
