# Supplementary figures and images for: Epigenetic regulation of EFEMP1 in prostate cancer: biological relevance and clinical potential
Source: J Cell Mol Med. 2014 Sep 11;18(11):2287–97. doi: 10.1111/jcmm.12394 (PMC4224561; doi:10.1111/jcmm.12394)

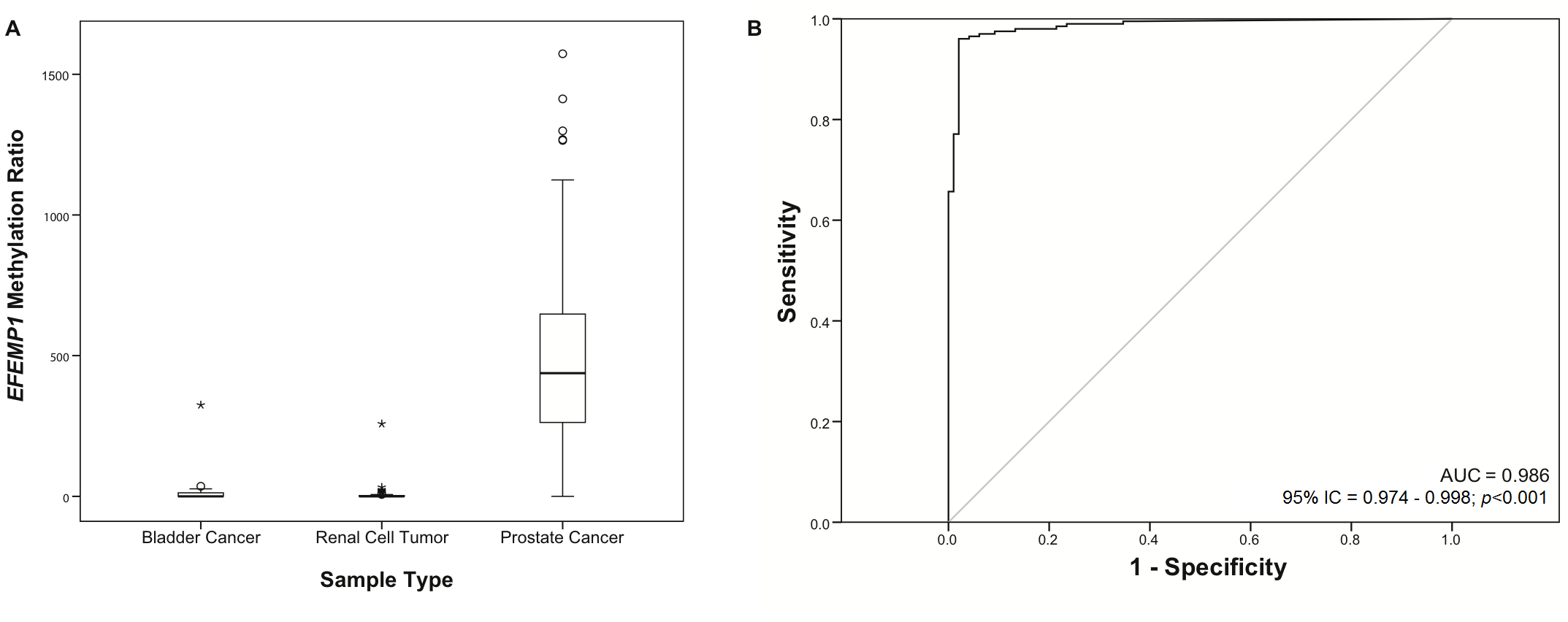

Supplement: Figure S1 — Distribution of EFEMP1 methylation levels in urological tumour samples and their performance as a prostate cancer biomarker. [file jcmm0018-2287-sd1.tiff]

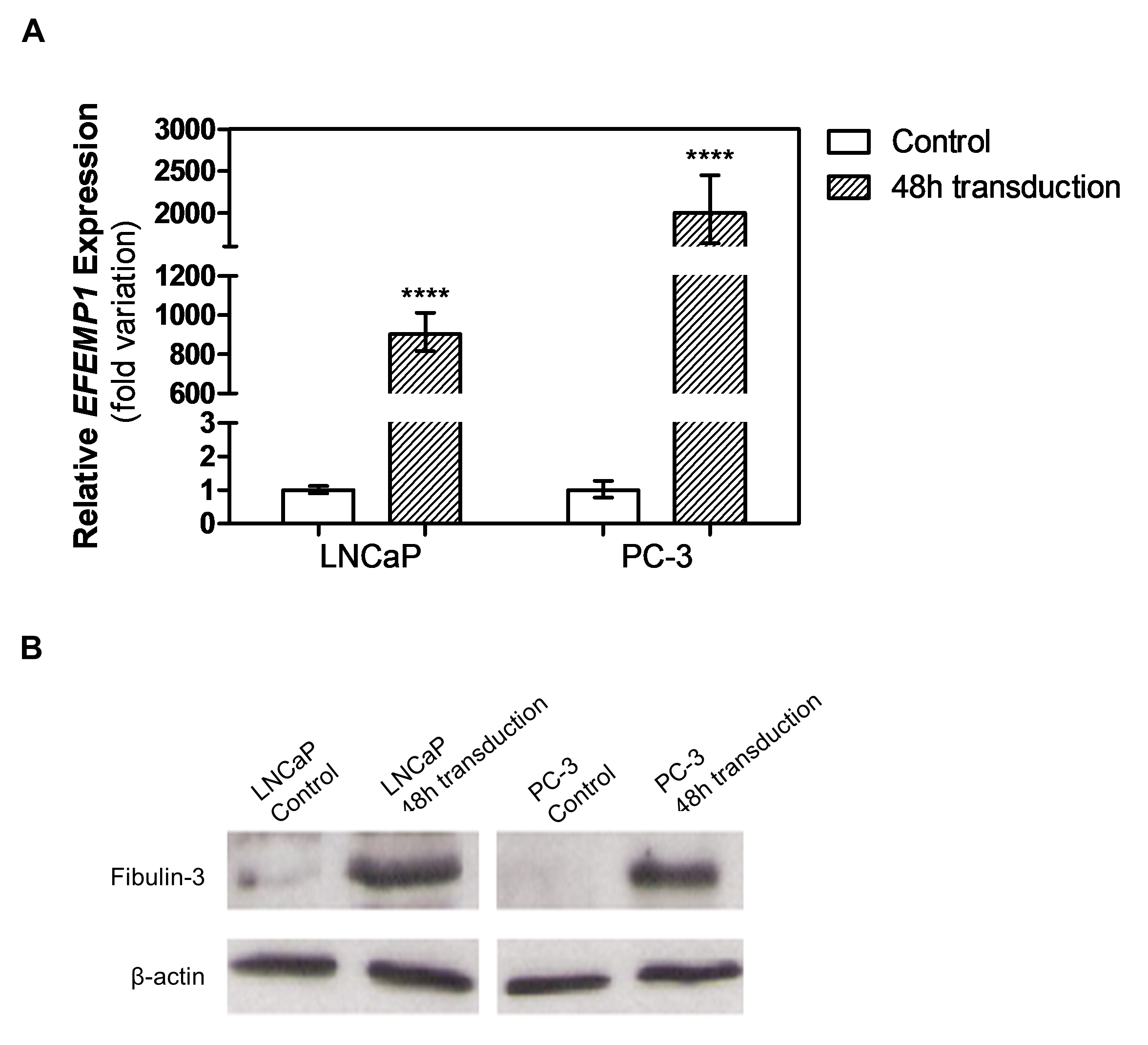

Supplement: Figure S2 — Validation of EFEMP1 transfection. [file jcmm0018-2287-sd2.tif]
