# Supplementary material for: Epigenetic regulation of EFEMP1 in prostate cancer: biological relevance and clinical potential
Source: J Cell Mol Med. 2014 Sep 11;18(11):2287–97. doi: 10.1111/jcmm.12394 (PMC4224561; doi:10.1111/jcmm.12394)
Supplement: Table S1 — Genes selected by microarray gene expression analysis as potential epigenetic biomarkers that were screened by MSP in this study. Table S2 Primers designed for qMSP and for ChIP assay. Table S3 Distribution of EFEMP1 methylation levels quantified by qMSP and methylation frequency in prostatic tissues. Table S4 Clinical and histopathological features of the patients and controls. Table S5 Diagnostic performance parameters of EFEMP1 methylation levels to distinguish NMT from PCa. Table S6 Distribution of EFEMP1 methylation levels quantified by qMSP and methylation frequency in other urological tumours. Table S7 Diagnostic performance parameters of EFEMP1 methylation levels to distinguish PCa from other urological tumours (BCa, RCT). [file jcmm0018-2287-sd3.docx]

**Supplementary Tables**

Supplementary Table I: Genes selected by microarray gene expression analysis as potential epigenetic biomarkers that were screened by MSP in this study**.**

|  | **Gene Symbol** | **Gene Name** |
| --- | --- | --- |
| 1 | *CHST2* | Carbohydrate (N-acetylglucosamine-6-O) Sulfotransferase 2 |
| 2 | *KCNH3* | Potassium voltage-gated channel, subfamily H (eag-related), member 3 |
| 3 | *UCHL1* | Ubiquitin carboxyl-terminal esterase L1 |
| **4** | ***EFEMP1*** | **EGF containing fibulin-like extracellular matrix protein 1** |
| 5 | *EPB41L3* | Erythrocyte membrane protein band 4.1-like 3 |
| 6 | *SERPINE2* | Serpin peptidase inhibitor, clade E (nexin, plasminogen activator inhibitor type 1), member 2 |
| 7 | *NEFH* | Neurofilament, heavy polypeptide |
| 8 | *BAI2* | Brain-specific angiogenesis inhibitor 2 |
| 9 | *CRISPLD2* | Cysteine-rich secretory protein LCCL domain containing 2 |
| 10 | *DEPDC7* | DEP domain containing 7 |
| 11 | *AHR* | Aryl hydrocarbon receptor |
| 12 | *NID2* | Nidogen 2 (osteonidogen) |
| 13 | *CACNA1G* | Calcium channel, voltage-dependent, T type, alpha 1G subunit |
| 14 | *MT1A* | Metallothionein 1A |
| 15 | *CHST11* | Carbohydrate (chondroitin 4) sulfotransferase 11 |
| 16 | *MXRA7* | Matrix-remodelling associated 7 |
| 17 | *RBP1* | Retinol binding protein 1, cellular |
| 18 | *PRKAG2* | Protein kinase, AMP-activated, gamma 2 non-catalytic subunit |
| 19 | *KCNH2* | Potassium voltage-gated channel, subfamily H (eag-related), member 2 |
| 20 | *GRK5* | G protein-coupled receptor kinase 5 |
| 21 | *DNER* | Delta/notch-like EGF repeat containing |
| 22 | *CERK* | Ceramide kinase |
| 23 | *PER3* | Period circadian clock 3 |

Supplementary Table II: Primers designed for qMSP and for ChIP assay

| **Technique** | **Designation Primer/Probe** | **Sequence** |
| --- | --- | --- |
| qMSP | EFEMP1 - Forward | 5’-TAGGGGATCGTCGCGTTAGT-3’ |
| qMSP | EFEMP1 - Reverse | 5’-AATCGCACCGCAACCC-3’ |
| qMSP | EFEMP1 - Probe | 5’-FAM™- GTCGGGTTAGGTCGTT–MGB™-NFQ-3’ |
| qMSP | ACTβ - Forward | 5’- TGGTGATGGAGGAGGTTTAGTAAGT- 3’ |
| qMSP | ACTβ - Reverse | 5’- AACCAATAAAACCTACTCCTCCCTTAA- 3’ |
| qMSP | ACTβ - Probe | 5’- FAM™ -ACCACCACCCAACACACAATAACAAACACA– TAMRA™- 3’ |
| ChIP | -2193bp (Forward) | 5’-ACAGACGTCTCTTCAACACACTG-3’ |
| ChIP | -2193bp (Reverse) | 5’–GGTCTCTCAAACCTCCCTTTATG-3’ |
| ChIP | -1583bp (Forward) | 5’-CCCCGCGAGTCTGGGAAACG-3’ |
| ChIP | -1583bp (Reverse) | 5’-CTGAGCCCAGCGTTGCGAGC-3’ |
| ChIP | -668bp (Forward) | 5’-GTGGAAATGCCACTTTGAGAG-3’ |
| ChIP | -668bp (Reverse) | 5’- CAAAACTCGGAGAGCAATCTTC-3’ |

Supplementary Table III: Distribution of *EFEMP1* methylation levels quantified by qMSP and methylation frequency in prostatic tissues

|  | ***EFEMP1* Methylation Ratio,** Median (IQR) | **Methylation Frequency,** n (%) | |
| --- | --- | --- | --- |
| **Prostate Samples** | | |  |
| NPT | 6.548 (3.379-26.148) | 1/15 (6.67%) | |
| BPH | 7.642 (4.124-11.389) | 1/32 (3.13%) | |
| HGPIN | 48.641 (16.545-152.979) | 37/73 (50.68%) | |
| PCa | 437.588 (262.364-647.650) | 193/201 (96.02%) | |

NPT - morphologically normal prostate tissue, BPH - benign prostatic hyperplasia, HGPIN - high grade prostatic intraepithelial neoplasia, PCa - prostate carcinoma, IQR - Interquartile range

Supplementary Table IV: Clinical and histopathological features of the patients and controls.

|  | **Prostate Samples** | | | | |
| --- | --- | --- | --- | --- | --- |
| **Clinicopathological Features** | | **NPT (n=15)** | **BPH (n=32)** | **HGPIN (n=73)** | **PCa (n=201)** |
| **Median Age**, *yrs* (range) | | 64 (45-80) | 68 (54-81) | 65 (49-75) | 64 (49-75) |
| **PSA** (ng/mL), median (range) | | *n.a.* | 4.70  (0.67-32.5) | 8.00  (2.66-17.70) | 8.10  (2.66-35.50) |
| **Pathological Stage,** *n* (%) | | | | | |
| pT2 | | *n.a.* | *n.a.* | 45 (62) | 113 (56) |
| pT3 | | *n.a.* | *n.a.* | 28 (38) | 88 (44) |
| **Gleason Score,** *n (%)* | | | | | |
| <7 | | *n.a.* | *n.a.* | 29 (40) | 68 (34) |
| =7 | | *n.a.* | *n.a.* | 42 (57) | 117 (58) |
| >7 | | *n.a.* | *n.a.* | 2 (3) | 16 (8) |

NPT - morphologically normal prostate tissue, BPH - benign prostatic hyperplasia, HGPIN - high grade prostatic intraepithelial neoplasia, PCa - prostate carcinoma, *n.a.* – not applicable

Supplementary Table V: Diagnostic performance parameters of *EFEMP1* methylation levels to distinguish NMT from PCa

| **Biomarker Parameters** | **Value (%)** |
| --- | --- |
| Sensitivity | 96.02% |
| Specificity | 97.87% |
| Positive Predictive Value | 99.48% |
| Negative Predictive Value | 85.19% |
| Accuracy | 96.37% |

Supplementary Table VI: Distribution of *EFEMP1* methylation levels quantified by qMSP and methylation frequency in other urological tumors

|  | ***EFEMP1* Methylation Ratio**  Median (IQR) | **Methylation Frequency**  n (%) | |
| --- | --- | --- | --- |
| **Other Urological Tumors** | | |  |
| Renal Cell Tumor | 0.000 (0.000-2.345) | 1/73 (1.4%) | |
| Bladder Cancer | 0.000 (0.000-12.699) | 1/24 (4.2%) | |

Supplementary Table VII: Diagnostic performance parameters of *EFEMP1* methylation levels to distinguish PCa from other urological tumors (BCa, RCT)

| **Biomarker Parameters** | **Value (%)** |
| --- | --- |
| Sensitivity | 96.02% |
| Specificity | 97.94% |
| Positive Predictive Value | 98.97% |
| Negative Predictive Value | 92.23% |
| Accuracy | 96.64% |
